# Longevity of Repair Versus Replacement of Partially Fractured Direct Composite Restorations in Permanent Teeth: A Systematic Review and Meta-Analysis

**DOI:** 10.7759/cureus.88307

**Published:** 2025-07-19

**Authors:** Mansi Vandekar, Gayatri Pendse, Nikita Toprani, Nikita Shitole, Priyanka Pannikar, Pooja Chadchan

**Affiliations:** 1 Department of Conservative Dentistry and Endodontics, DY Patil University School of Dentistry, Navi Mumbai, IND

**Keywords:** composite restoration, dental restoration, fractured restorations, restoration repair, restoration replacement

## Abstract

The present systematic review evaluates the comparative longevity of repaired versus replaced partially fractured direct composite restorations in permanent teeth. The decision to repair or replace a fractured restoration is a significant clinical consideration, influencing functional stability, esthetics, and preservation of tooth structure. Following the PRISMA guidelines, a comprehensive search of PubMed, Scopus, and Web of Science was conducted using predefined keywords. Inclusion criteria encompassed clinical and laboratory studies assessing the longevity, success rate, and failure parameters of repaired and replaced restorations. The methodological quality of observational studies was assessed using the Newcastle-Ottawa Scale, while interventional studies were evaluated using the Cochrane Risk of Bias (ROB-2) tool. A meta-analysis was performed to compare restoration longevity based on parameters such as marginal adaptation, anatomic form, surface roughness, marginal staining, and secondary caries. Nine studies published between 2006 and 2018 were included, with follow-up periods ranging from 2 to 15 years. The meta-analysis revealed that repaired restorations demonstrated superior marginal adaptation (RR = 0.47; p < 0.05) and lower secondary caries incidence (RR = 0.72; p > 0.05), favouring repair over replacement. However, no significant differences were found for anatomic form, surface roughness, or marginal staining. While the methodological quality varied, studies consistently highlighted the benefits of repair in preserving tooth structure and minimizing treatment invasiveness. Repaired composite restorations offer comparable longevity to complete replacement while conserving more tooth structure and reducing patient discomfort. The findings advocate for prioritizing repair as a conservative approach before opting for full restoration replacement. Further longitudinal studies with standardized protocols are needed to optimize restorative strategies.

## Introduction and background

Direct resin-based composite (RBC) restorations have become a cornerstone of contemporary restorative dentistry, offering a versatile and aesthetic solution for managing teeth compromised by caries, trauma, or mechanical degradation. Their ability to bond micromechanically and chemically to enamel and dentin, combined with their conservative nature and improved material properties, has made them the material of choice for both initial restorations and interventions involving partially fractured or defective restorations [1].

Fracture or failure of restorations may arise from occlusal forces, secondary caries, or marginal degradation over time [2]. When this occurs, clinicians must decide whether to completely replace the restoration or opt for a more conservative repair approach. Repair involves selectively removing the defective portion while preserving the intact structure, followed by surface treatment and placement of a new composite. Replacement, on the other hand, entails full removal and replacement of the existing restoration. While complete replacement enables a comprehensive assessment of the cavity and substrate, it also involves increased removal of sound tooth tissue, elevated chair time, and greater biological and financial costs. Repair, in contrast, aligns with the principles of minimally invasive dentistry, aiming to preserve natural tooth structure, reduce patient discomfort, and maintain restoration longevity whenever feasible [3].

Despite these theoretical advantages, the clinical decision between repair and replacement remains nuanced. While various protocols exist for composite repair, including surface roughening, etching, and the use of intermediate adhesives, questions persist about the long-term success and durability of repaired restorations. Several studies have attempted to address these concerns, but the clinical evidence remains fragmented.

A number of recent systematic reviews have contributed to this discussion. Mendes et al. (2022) conducted a systematic review and meta-analysis comparing the failure risk of repaired and replaced defective restorations (including both amalgam and composites) and concluded that there was no statistically significant difference in failure risk, although the certainty of evidence was very low due to methodological limitations [4]. Al Rabiah et al. (2021) focused on the bond strength and adhesion potential of repaired RBCs but did not address clinical longevity or failure outcomes. They also highlighted the need for high-quality randomized trials due to inconsistencies across protocols and outcome measures [5]. 

These studies underscore both the promise and the limitations of current knowledge. Importantly, no prior review has comprehensively and exclusively synthesized clinical outcome data comparing repaired and replaced direct composite restorations in permanent teeth. Previous reviews have either combined materials, focused on laboratory outcomes, or relied on retrospective data without meta-analytic synthesis.

The present systematic review was thus undertaken to fill this gap by evaluating and comparing the clinical longevity, failure rates, and overall performance of repaired versus replaced resin composite restorations in permanent teeth. The review aims to generate evidence-based, clinically relevant recommendations that support minimally invasive, patient-centered restorative care by including a meta-analysis and focusing exclusively on clinical outcomes such as marginal adaptation, anatomical form, secondary caries, and restoration success or failure.

## Review

Methodology

The present systematic review was conducted following the guidelines of the Preferred Reporting Items for Systematic Reviews and Meta-Analyses (PRISMA) 2020 [6]. The protocol was registered in the PROSPERO international prospective register of systematic reviews under the reference ID (CRD42023469157). The primary research question framed for this review was: “What is the comparative longevity of repaired and replaced partially fractured permanent restorations with direct composite restorations in permanent teeth?”

Search Strategy

A comprehensive and systematic literature search was conducted to identify relevant studies comparing repaired and replaced partially fractured restorations using direct composite resin in permanent teeth. The electronic databases searched included PubMed/MEDLINE (via NCBI platform), Scopus (via Elsevier interface), and the Web of Science Core Collection (via Clarivate Analytics interface). All databases were searched from their inception until March 30, 2024, which was the final date of the search for all electronic sources.

The search strategy utilized a combination of medical subject headings (MeSH) and free-text terms. Keywords included: "composite restoration," "repair," "replacement," "fractured restorations," and "longevity." Boolean operators such as “AND” and “OR” were used to appropriately combine search terms across all databases. Filters were applied to limit search results to human studies published in the English language.

No restrictions were imposed on publication date, geographic location, or publication status. Titles and abstracts of retrieved articles were exported into a reference management software (EndNote, Clarivate, London, UK) for screening and de-duplication.

In addition to database searches, reference lists of all included full-text articles and previous systematic reviews on similar topics were manually screened to identify additional eligible studies. Forward and backward citation tracking was also performed on included articles using the Web of Science and Google Scholar platforms. This citation search was completed on April 2, 2024, and included cited references and articles citing the included studies. No trial registries, manufacturer databases, conference proceedings, or regulatory repositories were searched. Additionally, no individuals or organizations were contacted for additional study identification.

Inclusion and Exclusion Criteria

Inclusion criteria were defined to select studies that directly compared the longevity, success rate, or failure rate of repaired versus replaced partially fractured restorations in permanent teeth using direct composite materials. Both randomized controlled trials and clinical observational studies (prospective or retrospective in design) were included. Eligible studies were required to report outcome parameters related to restoration durability, including failure rates, retention, marginal adaptation, anatomical form, marginal staining, secondary caries, and overall esthetics. Only articles available as full-text and published in peer-reviewed journals in English were included.

Exclusion criteria encompassed in vitro laboratory studies, animal studies, case reports, narrative reviews, conference abstracts, and articles not comparing repair and replacement interventions. Studies involving restorative materials other than resin-based composites (e.g., amalgam, ceramics) were excluded, as were those not reporting on clinically relevant longevity or durability outcomes. The PICOS framework used for determining the eligibility of the articles in the present systematic review is listed in Table 1 [6].

**Table 1:** PICOS Framework used for the selection of studies in the present systematic review

PICOS Component	Inclusion Criteria	Exclusion Criteria
Population	Studies involving permanent teeth with partially fractured restorations	Studies on primary teeth, fully fractured restorations, or intact restorations
Intervention	Repair of defective or partially fractured composite restorations using direct resin composites	Interventions using materials other than composites (e.g., amalgam, ceramic); full coverage restorations like crowns
Comparison	Replacement of partially fractured composite restorations with new direct resin composite restorations	Comparisons with indirect restorations, no comparison group, or restoration types not involving composite resin
Outcomes	Reports on longevity, failure rates, marginal adaptation, anatomic form, surface roughness, marginal staining, or secondary caries	Studies lacking quantitative outcome data, no reporting on restoration performance or durability
Study Design	Randomized controlled trials (RCTs), non-randomized clinical studies, prospective/retrospective observational studies, practice-based research	Review articles, case reports, conference abstracts, in vitro studies, expert opinions

Study Selection

The study selection process was conducted in two stages. Initially, two independent reviewers (MV and NS) screened the titles and abstracts of all retrieved records. Articles that appeared to meet the inclusion criteria or required further evaluation were retrieved in full text. Full-text screening was conducted independently by both reviewers using predefined eligibility criteria. Any disagreements between the two reviewers regarding study inclusion were resolved through discussion and, when necessary, consultation with a third reviewer (GP). Duplicate publications were identified and removed during the screening process.

Data Extraction

 A structured and pre-tested data extraction form was used to collect relevant information from the included studies. Data were independently extracted by two reviewers and cross-verified. Extracted data included the following: study characteristics (authors, year of publication, country of origin, and study design), participant details (sample size, patient age or age range), intervention details (type of restoration, cavity classification, repair or replacement method), outcome measures (failure rate, success rate, marginal adaptation, anatomical form, surface texture, esthetics, secondary caries, and other indicators of clinical longevity), and follow-up durations. Any missing or unclear data were noted, and attempts were made to clarify through the article content.

Assessment of Methodological Quality

The methodological quality of the included studies was assessed using validated risk of bias tools appropriate to the study design. For observational studies, quality was assessed using the Newcastle-Ottawa Scale (NOS) [7], which evaluates studies across three domains: selection, comparability, and outcome. Each study was assigned a numeric score based on the number of stars earned in each domain.

For randomized controlled trials (RCTs), the Cochrane Risk of Bias 2 (ROB-2) tool was employed [8]. This tool evaluates five domains of potential bias: randomization process, deviations from intended interventions, missing outcome data, measurement of the outcome, and selection of the reported result. A risk of bias summary figure and applicability concerns graph were generated using Review Manager (RevMan) software version 5.3.

Data Synthesis and Statistical Analysis

The data synthesis process involved a qualitative summary of study findings, followed by a meta-analysis where appropriate. Meta-analysis was conducted only when included studies demonstrated sufficient homogeneity in study design, population, and outcome measures. For dichotomous outcomes (e.g., success or failure of restorations), the risk ratio (RR) with 95% confidence intervals (CI) was calculated.

The choice between a fixed-effects or random-effects model was guided by the degree of statistical heterogeneity, assessed using Cochran’s Q-test and the I² statistic. An I² value above 50% or a p-value below 0.1 was interpreted as significant heterogeneity, in which case a random-effects model was used. If heterogeneity was low (I² ≤ 24% and p > 0.1), a fixed-effects model was adopted. To evaluate potential publication bias, Begg’s funnel plot was generated and visually assessed for asymmetry. All statistical analyses were performed using RevMan software version 5.3 (The Cochrane Collaboration, London, UK), and the threshold for statistical significance was set at p < 0.05.

Results

**Figure 1:**
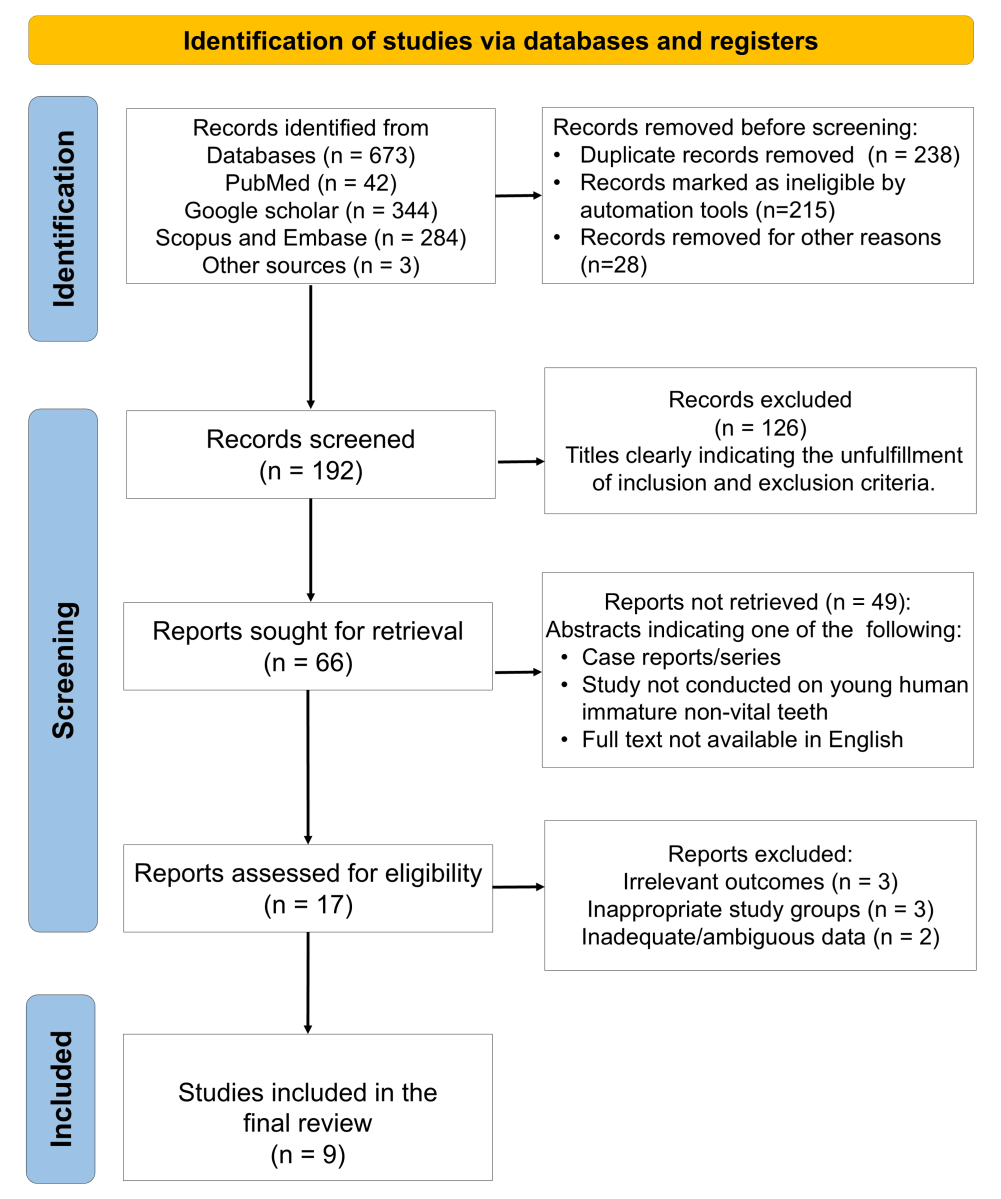
PRISMA flow diagram indicating the selection process of the articles for data synthesis in the present systematic review.

A total of 673 articles were identified from database searching, out of which 192 articles were screened following removal of duplicates and those marked ineligible or removed for other reasons. After screening of abstracts and full texts, nine studies were identified to be eligible (Figure 1) that were published across the period 2006 to 2018 [9-17]. The data concerning the characteristics and outcomes of the studies extracted from the included articles are summarized in Table 2.

**Table 2:** Characteristics and outcomes of the studies included in the present systematic review RBC: resin-based composite; Am: amalgam; Rep: repair; Repl: replacement; MI: minimally invasive; NR: not reported; USPHS: United States Public Health Service criteria; y: years.

Sr. No.	Author (Year)	Country	Study Designs	n	Age	Teeth selection	Groups	Assignment	Cavities	Inclusion	Exclusion	Criteria	Follow-up	Isolation	Repair	Replacement	Comparison	Conclusive findings
1	Gordan et al. (2006) [9]	USA (FL)	Longitudinal clinical study	88	27–77 (55)	Defective RBC restorations	Rep; Seal; Resurf; Repl; Control	Random	Cl III–V	Adults; Bravo defects	Contra-indications; Charlie lesions	USPHS	2 y	Rubber dam	Partial removal + RBC	Full replacement RBC	Resurfacing inferior; repair best	Repair conservative; replacement unjustified
2	Moncada et al. (2008) [10]	USA	Longitudinal clinical trial	271	18–80 (26.6)	Defective Am/RBC	Rep; Seal; Refurb; Repl; Control	Random	—	Marginally deficient Am/RBC	Systemic contra-indications	USPHS	2 y	Rubber dam	Bur clean-up + sealant	Full replacement RBC	Repair ≈ replacement survival	Repair/refurbish extend restoration life
3	Gordan et al. (2009) [11]	USA	Longitudinal clinical study	88	27–78 (57)	Defective restorations	Rep; Seal; Resurf; Repl; Control	Random	—	Adults; defective restos	Contra-indications	Mod USPHS	0.5–7 y	Rubber dam	Localized removal + RBC	Full replacement RBC	Repair ≥ replacement; control worst	MI repair preferred for longevity
4	Moncada et al. (2009) [12]	USA	Longitudinal clinical trial	66	18–80 (26.5)	Am/RBC defects	Seal; Refurb; Rep; Repl	Criteria-based	Cl I/II	≥ 20 teeth; marginal defects	Contra-indications	USPHS	1–3 y	Rubber dam	Partial removal + RBC	Total replacement	Only surface lustre differed	Repair adequate for Cl I/II RBC
5	Fernández et al. (2011) [13]	Chile	Longitudinal clinical study	66	18–80 (26.5)	Marginal Am/RBC defects	Seal; Refurb; Rep; Repl	Criteria-based	—	Marginal defects	Contra-indications	USPHS	—	Rubber dam	Bur edge repair + seal	Total replacement	Repair poorest marginal fit	Sealing alone inadequate; thorough repair needed
6	Fernández et al. (2015) [14]	Chile	Longitudinal clinical trial	28	18–80	Localized marginal defects	Rep; Repl	Random	Cl I/II	Local marginal/anatomical defects	Contra-indications	Mod USPHS	10 y	Rubber dam	Bur + sealant repair	Full replacement	No difference over 10 y	Repair conserves tooth; performs similarly long-term
7	van de Sande et al., 2019 [15]	Brazil	Longitudinal clinical study	634	NR	Restorations needing revision	Rep; Repl	Defect-extent	Cl III/IV; veneers	Revision cases with opposing/adjacent teeth	Missing data	Defect class	15 y	Varied	Local removal + RBC overlay	Full replacement (Am/RBC)	Repair outcomes equivalent	Repair suitable for Cl III/IV & veneers
8	Estay et al. (2018) [16]	Chile	Longitudinal clinical study	167	18–80 (26.4)	Localized marginal defects	Rep; Repl; Pos ctrl	Random	—	Load-bearing Am/RBC restos	Contra-indications	Mod USPHS	12 y	Rubber dam	Spot repair + RBC patch	Full replacement	12 y: no clinical difference; repair cheaper	Repair cost-effective and effective
9	Kanzow and Wiegand, 2020 [17]	Germany	Retrospective Study	880	mean 38.8	Composite restorations ≥ 2 y	Rep; Repl	Retrospective	None	Composite restorations with ≥ 2 y records	Incomplete data	NR	10 y	NR	NR	NR	10 y success: 43 % repair vs 49 % replacement	Repairs improve survival; endorse MI strategy

Of these, four were conducted in Florida, USA, three were conducted in Chile, and one study each was conducted in Brazil and Germany, respectively. The sample size of the studies ranged from 66 to 880. Most of the studies included a sample population of ages ranging from 18 to 80 years, while two studies included the age group of 27 to 77 years. All studies examined resin-based composite (RBC) restorations with clinical deviations such as marginal deficiencies and secondary caries, graded using United States Public Health Service criteria (USPHS) or USPHS/Ryge criteria [18,19].

The authors divided the included teeth into treatment groups: marginal sealing, refurbishment, repair, replacement, and untreated teeth, which served as negative control. The assignment of samples to these respective groups was performed randomly in five studies [9,10,11,14,16] and on the basis of certain criteria in three studies [12,13,15]. The specifications of the cavity designs vastly affect the restorations in numerous ways, including mechanical properties, bonding, and displacement, to name a few. Although four studies specified cavity classifications, two focusing on Class I and II [12,14], and two on Class III, IV, and V [9,15], none of the included studies conducted subgroup analyses or reported differential outcomes based on cavity type. As such, the influence of caries classification on the longevity or clinical performance of repaired versus replaced restorations could not be quantitatively assessed in this review.

The authors used varied inclusion criteria which included the presence of more than 20 teeth, restorations in functional occlusion with an opposing natural tooth, asymptomatic restored tooth, at least one proximal contact area with a neighboring tooth, patients with marginal deficiencies in the restorations, at least one Bravo rating but no Charlie rating in any of the clinical characteristics observed in the study, older than 18 years old [9-17]. These criteria overlapped to different extents across the various studies.

Likewise, the exclusion criteria also varied and overlapped, which included contraindications for regular dental treatment according to their medical history, esthetic demands that could not be resolved by the alternative treatments, xerostomia, or were receiving treatment with medications that significantly reduced salivary flow, psychiatric or physical pathologies that interfered with oral hygiene and patients at an extremely high risk of developing caries, composite and amalgam restorations with localized defects by secondary caries or marginal defects greater than 3 mm and located and/or in the proximal surfaces, clinical judgment that repair was not indicated in resin or amalgam restorations and high-caries-risk patients and defects greater than 3 mm [9-16]. Kanzow and Wiegand (2020) excluded restorations with missing information (for instance, no information regarding restored tooth/surfaces available) and restorations with interventions after less than four weeks. The follow-up period ranged from 2 to 15 years [17].

The restorations in all the studies were performed under rubber dam isolation [9-17]. The protocol for repair of restorations was roughly similar, which involved removing the defective portion of the RBC by using a round carbide bur, acid etching the preparation and the remaining composite with 35% phosphoric acid, application of a resin-based bonding system, and restoration with RBC. The replacement procedure involved removal of the defective restoration, removal of caries, cavity preparation, and restoration with RBC [9-17].

Moncada et al. (2008) found that the repaired restorations showed improvements in all parameters, but they were significant only in anatomy and marginal stain, whereas in those replaced, all parameters, except for marginal stain, improved significantly [10]. With the exception of Gordan et al. (2009), who found lower failure rates in repaired restorations, all the other authors found that although the number of failures in repaired restorations was lower than in the replaced ones, the numbers were not significant [11]. Thus, they recommended that dentists should attempt minimally invasive procedures or conservative procedures such as repair, refurbishment, or sealing before proceeding to complete the replacement of restorations.

Methodological Quality of Included Studies

**Figure 2:**
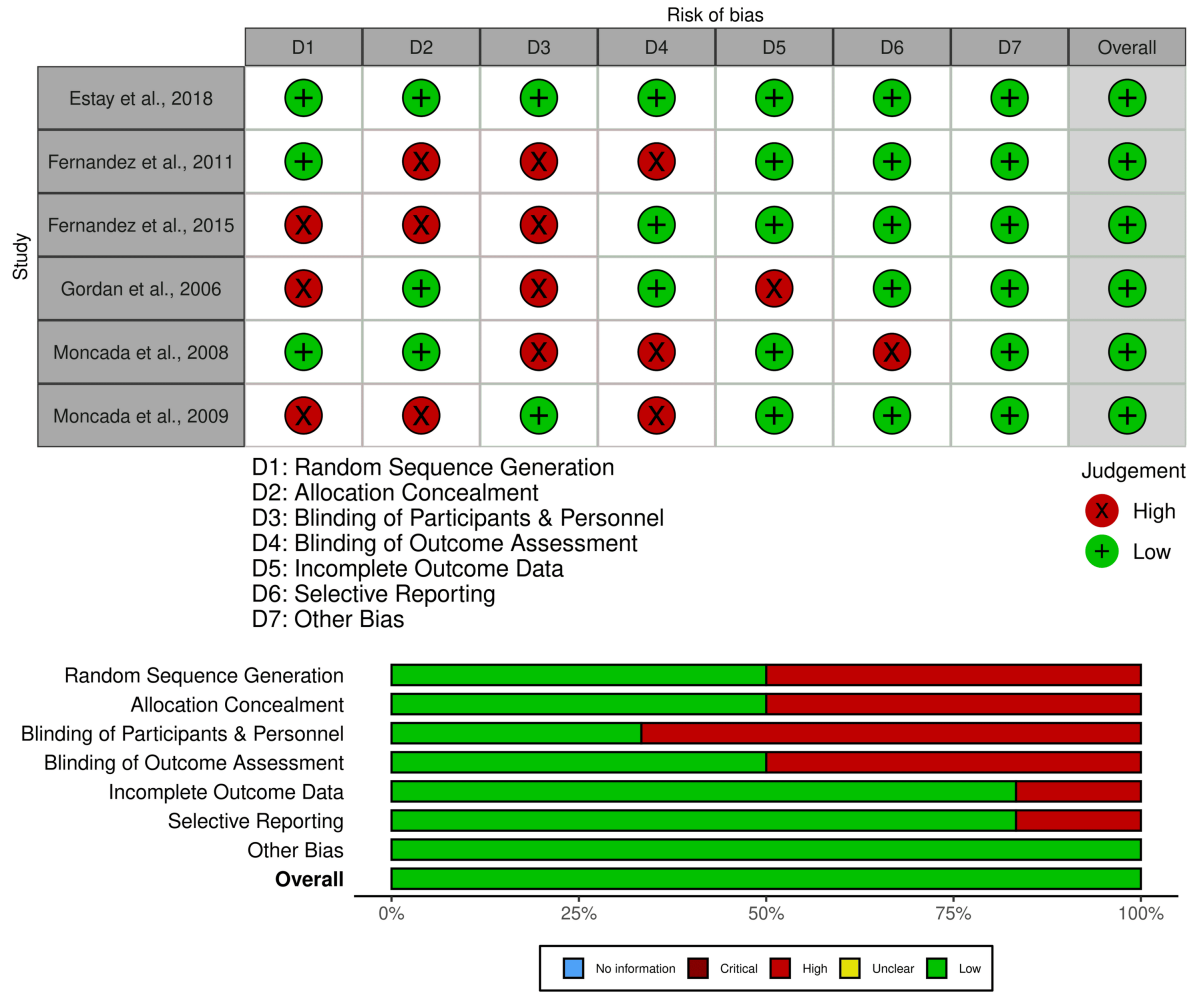
Risk of bias in the individual studies and summary of the clinical studies included in the present systematic review Gordan et al. [9], Moncada et al. [10, 12], Fernández et al. [13, 14], Estay et al. [16]

All the included studies were largely comparable in methodological quality. All the included studies had a moderate to high risk of bias in all the respective domains. The highest risk of bias was seen for blinding of participants and personnel (performance bias), followed by random sequence generation (selection bias), allocation concealment (selection bias), and blinding of outcome assessment (detection bias). Among the included studies, Estay et al. (2018) reported the lowest risk of bias, while other studies reported a moderate to low risk of bias with respect to other domains [16]. Domains of incomplete outcome data (attrition bias), followed by selective reporting (reporting bias), and other biases, were given the lowest risk of bias by included studies. The risk of bias in included studies assessed using the Cochrane ROB-2 tool is depicted in Figure 2.

Among the included studies, only one study (van de Sande et al., 2019) reached the maximum score of the Newcastle-Ottawa scale [15]. For selection bias, two studies (van de Sande et al., 2019; Kanzow and Wiegand, 2020) reached the maximum score, while for the comparability outcome, two studies (Gordan et al., 2009; van de Sande et al., 2019) reached the maximum score, and for exposure, two studies (van de Sande et al., 2019; Kanzow and Wiegand, 2020) reported maximum score and were considered to have the highest level of quality with an estimated low risk of bias [11,15,17]. The study had a moderate to low risk of bias, with the overall quality of the study being good. The risk of bias of the included studies assessed using the Newcastle-Ottawa scale is depicted in Table 3.

**Table 3:** Risk of bias in the retrospective study assessed using the Newcastle-Ottawa scale Each asterisk (*) corresponds to one point.

Author, year	Selection (max = 4)	Comparability (max = 2)	Exposure (max = 3)	Overall quality score (max = 9)
Gordan et al., 2009[11]	**	**	**	6
van de Sande et al., 2019 [15]	****	**	***	9
Kanzow and Wiegand, 2020 [17]	****	*	***	8

Meta-Analysis

The risk ratio (RR) was utilized as the summary effect measure for dichotomous variables. The aim was to compare the clinical longevity of repaired versus replaced partially fractured composite restorations in permanent teeth based on key criteria: marginal adaptation, anatomic form, surface roughness, marginal staining, and secondary caries.

Marginal adaptation

**Figure 3:**
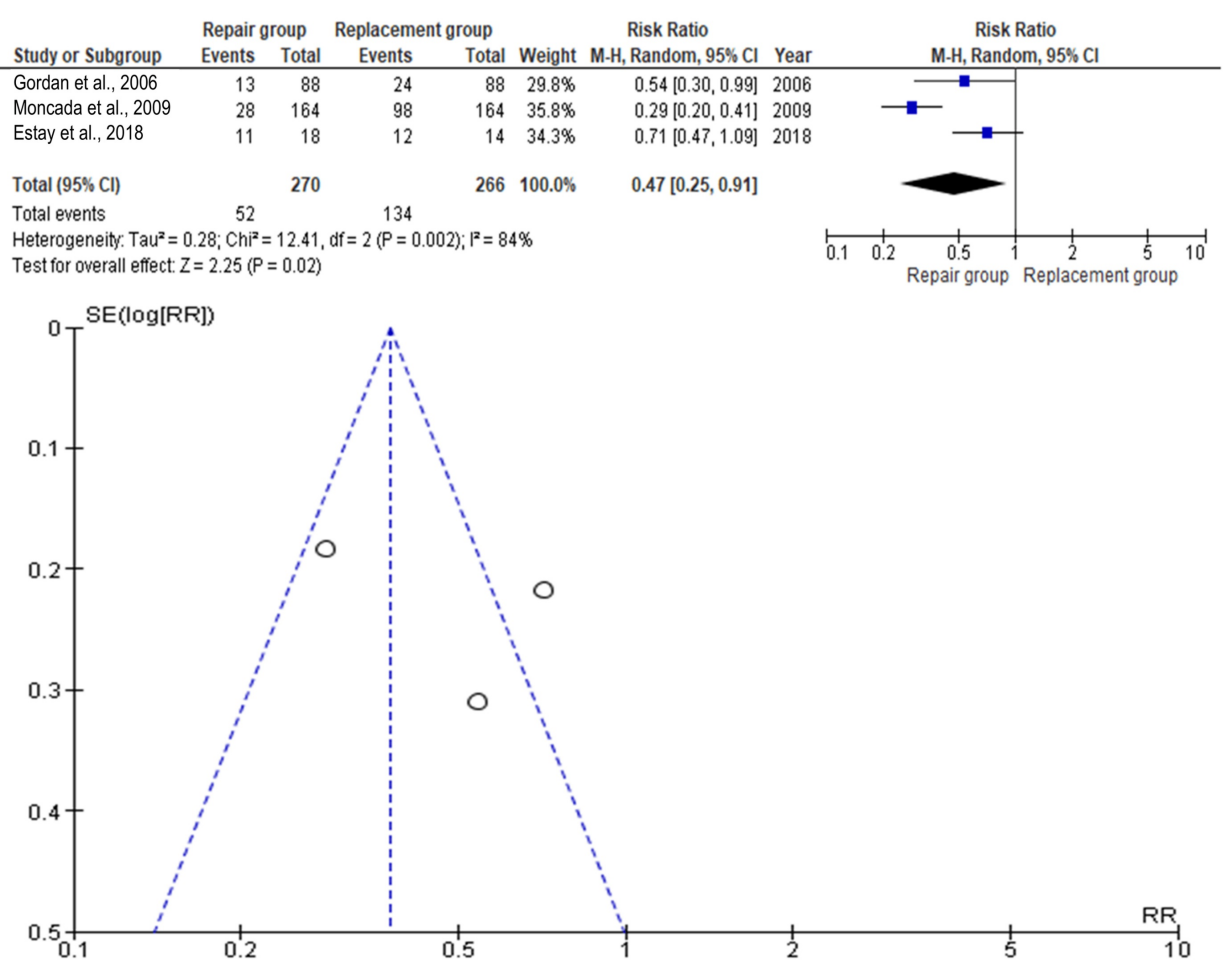
Forest plot and funnel plot of the included studies with regard to marginal adaptation Gordan et al. [9], Moncada et al. [12],  Estay et al. [16]

Three studies encompassing a total of 536 teeth (repair: n=270; replacement: n=266) were analyzed for marginal adaptation. As illustrated in Figure 3, the pooled risk ratio was 0.47 (95% CI: 0.25-0.91), significantly favoring the repair group (p<0.05). This indicates that the likelihood of favorable marginal adaptation was higher with repair than replacement. The study by Moncada et al. (2009) contributed the greatest statistical weight, while Gordan et al. (2006) contributed the least [9,12]. The funnel plot showed noticeable asymmetry, suggesting potential publication bias.

Anatomic Form

**Figure 4:**
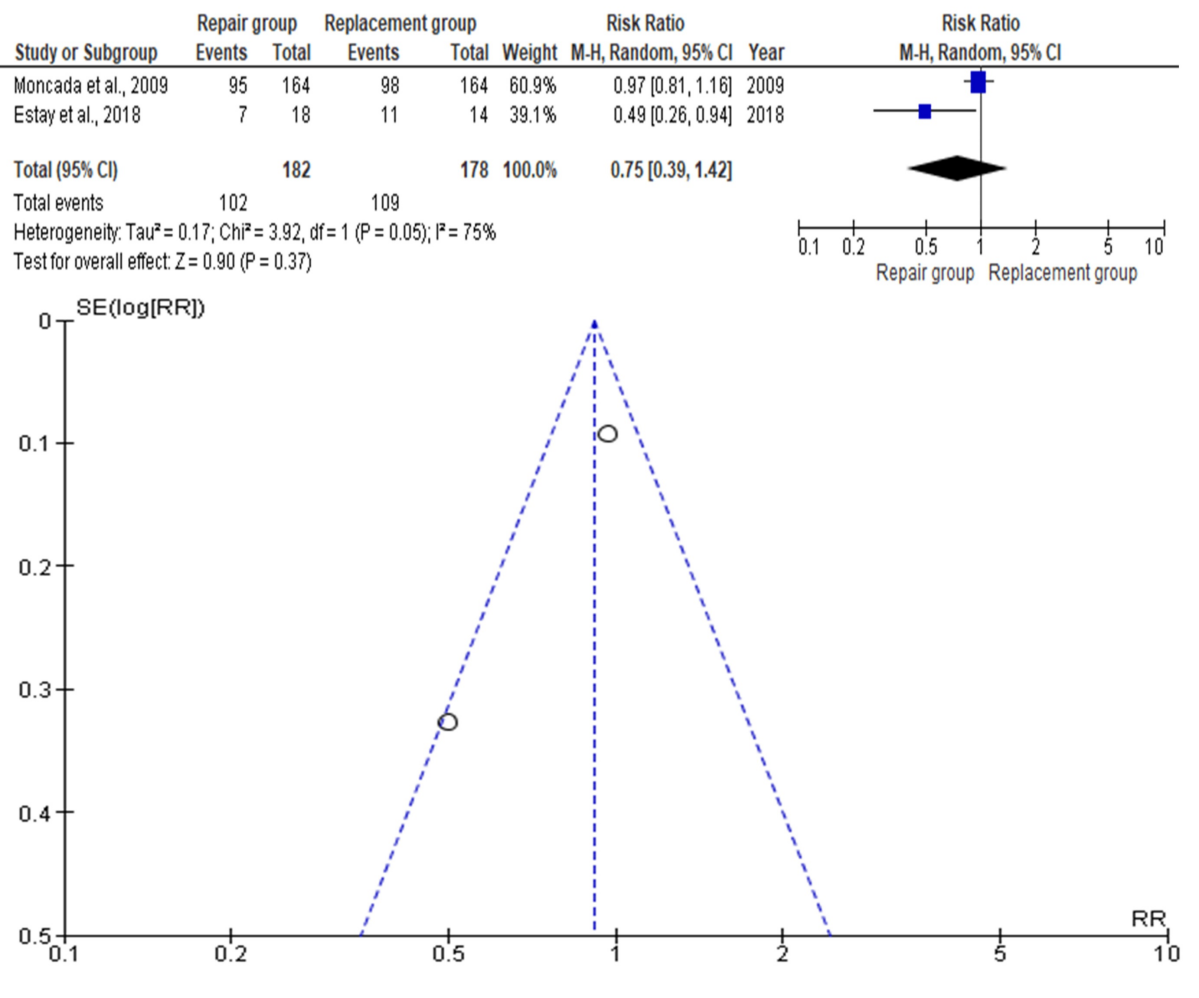
Forest plot and funnel plot of the included studies with regard to anatomic form Moncada et al. [12],  Estay et al. [16]

Two studies, including 360 teeth (repair: n=182; replacement: n=178), were included for evaluating anatomic form. The combined RR was 0.75 (95% CI: 0.39-1.42), as shown in Figure 4, favoring the repair group, although the difference was not statistically significant (p>0.05). Moncada et al. (2009) had the highest weight, while Estay et al. (2018) had the lowest [12,16]. The funnel plot indicated no significant asymmetry, suggesting a low risk of publication bias.

Surface Roughness

**Figure 5:**
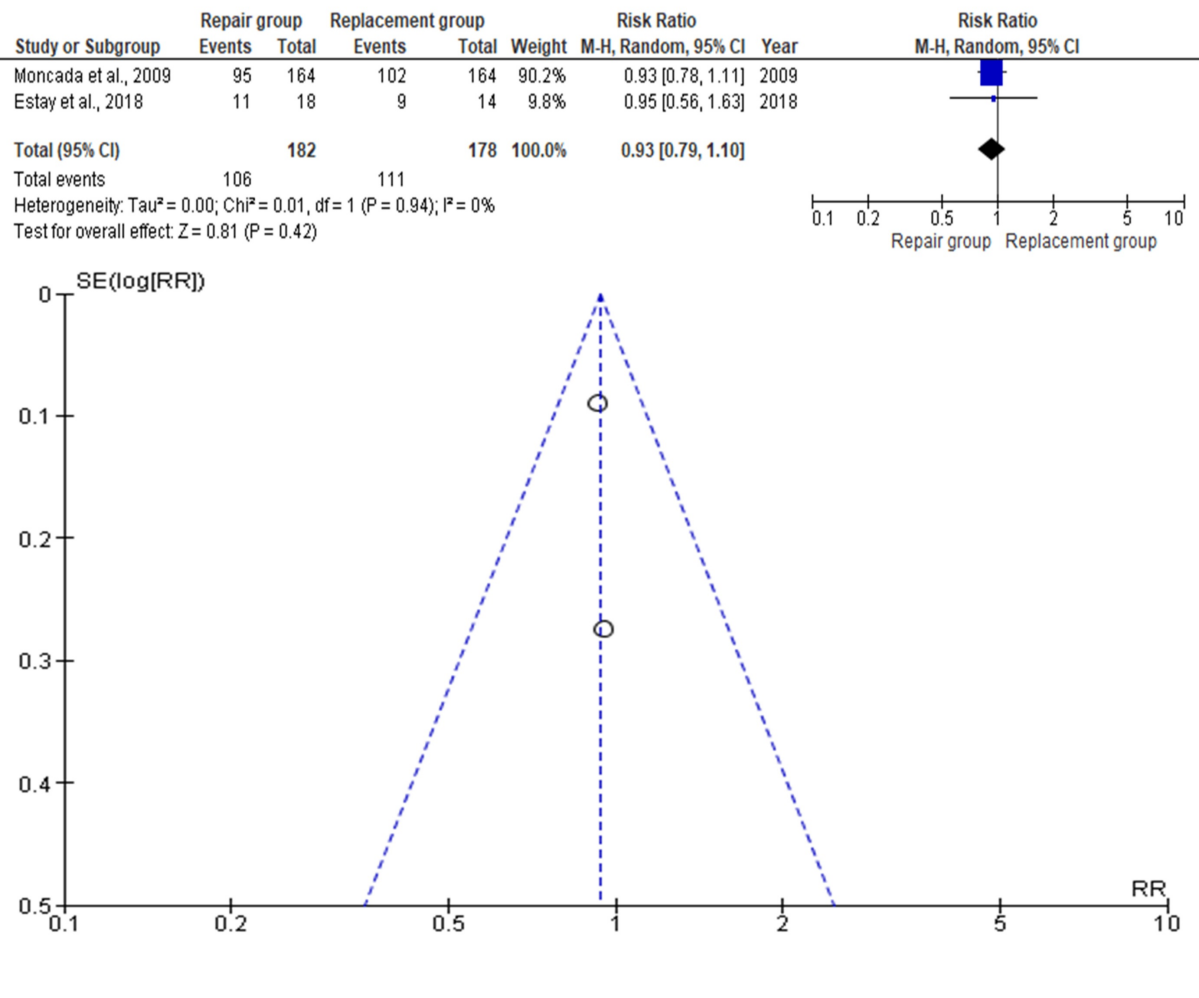
Forest plot and funnel plot of the included studies with regard to surface roughness Moncada et al. [12], Estay et al. [16]

Surface roughness was assessed in 360 teeth across two studies (repair: n=182; replacement: n=178). As depicted in Figure 5, the pooled RR was 0.93 (95% CI: 0.79-1.10), indicating a slight, non-significant advantage for the repair group (p>0.05). Moncada et al. (2009) had the highest study weight, and Estay et al. (2018) the lowest [12,16]. No asymmetry was detected in the funnel plot, implying minimal publication bias.

Marginal Staining

**Figure 6:**
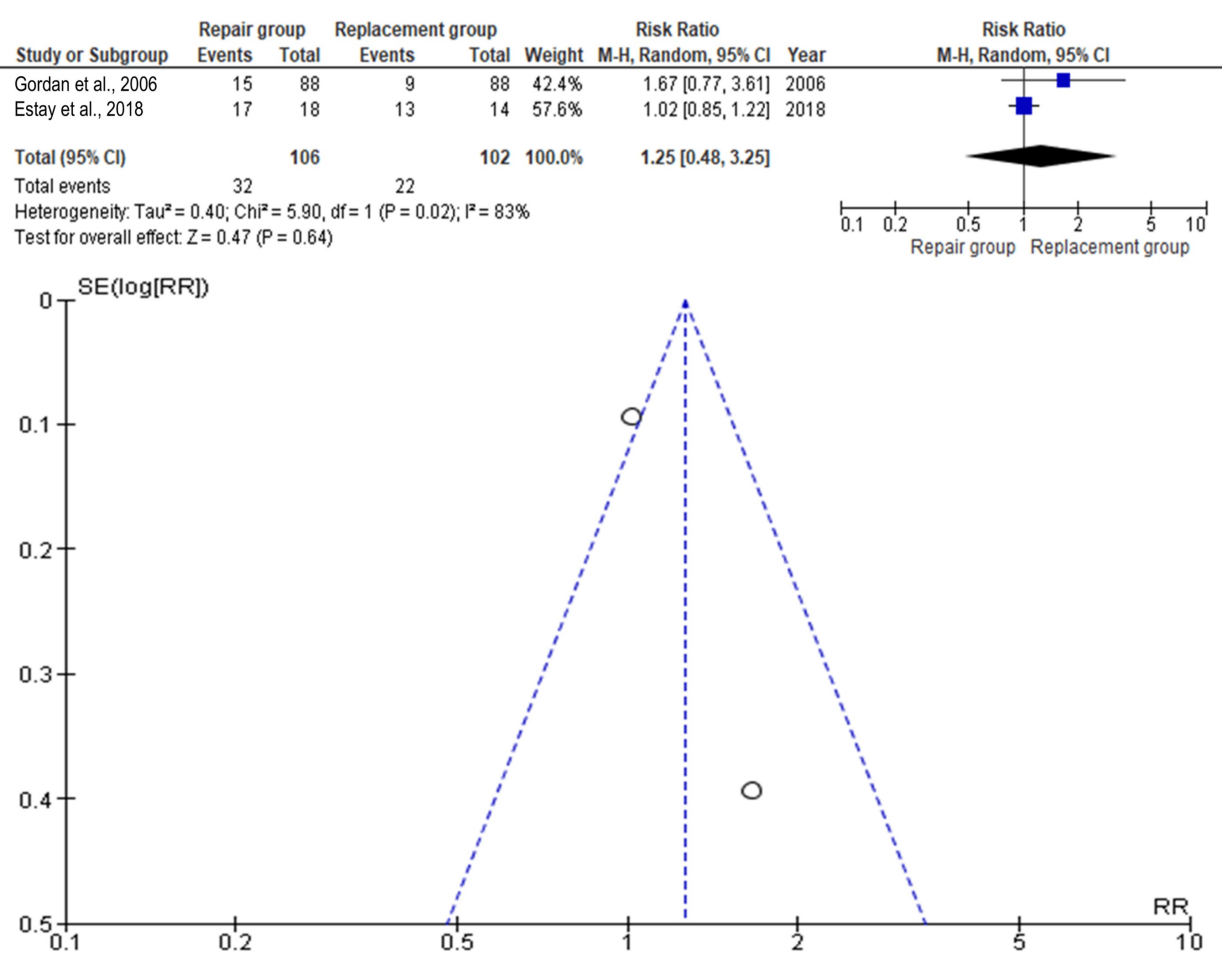
Forest plot and funnel plot of the included studies with regard to marginal staining Gordan et al. [9], Estay et al. [16]

Marginal staining outcomes were reported in two studies comprising 210 teeth (repair: n=106; replacement: n=102). As shown in Figure 6, the RR was 1.25 (95% CI: 0.48-3.25), with the replacement group showing higher rates, although not statistically significant (p>0.05). Estay et al. (2018) contributed the most weight, while Gordan et al. (2006) contributed the least [9,16].

Secondary Caries

**Figure 7:**
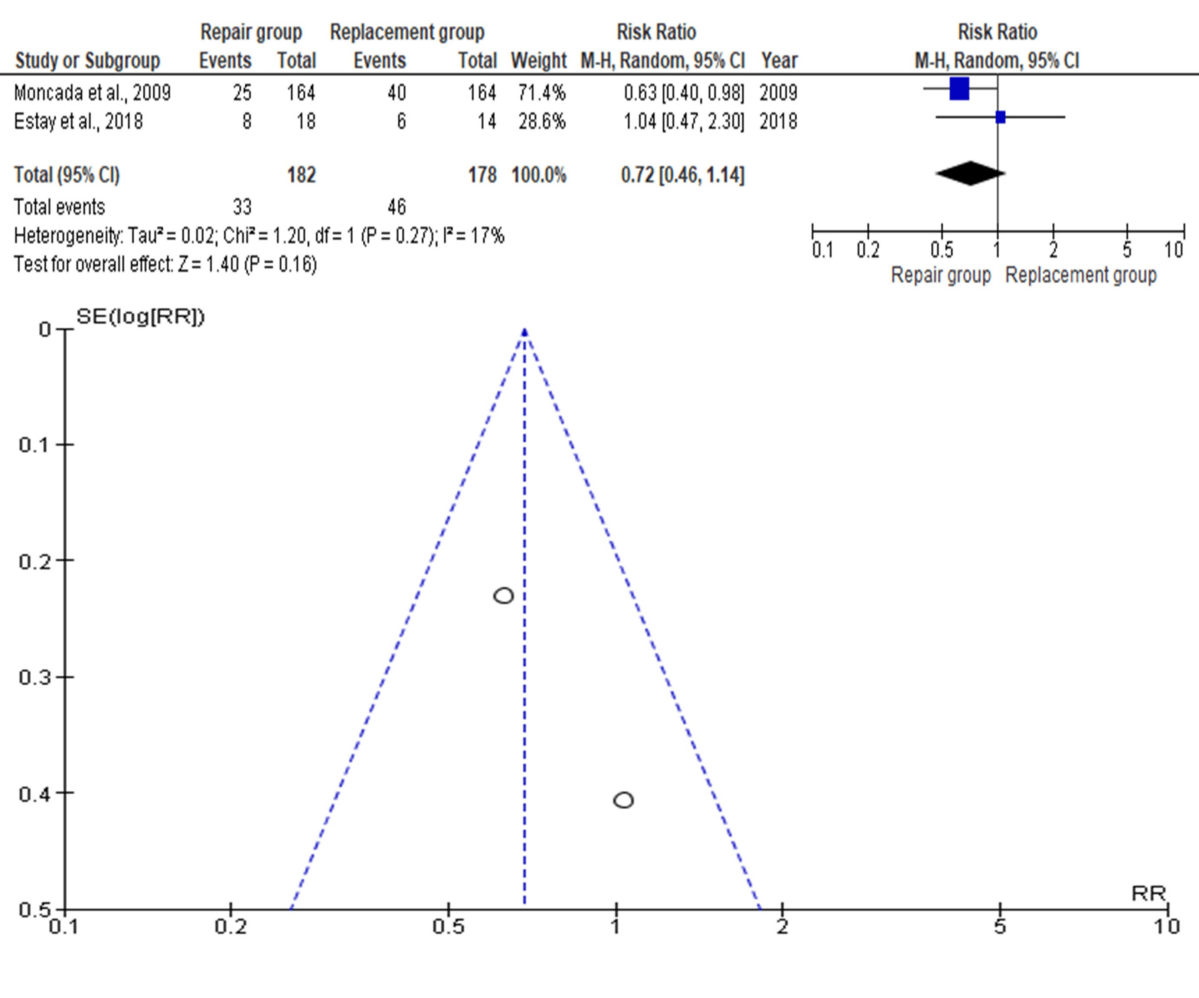
Forest plot and funnel plot of the included studies with regard to secondary caries Moncada et al. [12],  Estay et al. [16]

Two studies involving 360 teeth (repair: n=182; replacement: n=178) reported data on secondary caries. The pooled RR was 0.72 (95% CI: 0.46-1.14), indicating a favorable trend toward the repair group, though the difference was not statistically significant (p>0.05) as shown in Figure 7. Moncada et al. (2009) held the highest weight, with Estay et al. (2018) contributing the least [12,16]. 

Discussion

The findings of this systematic review provide valuable insights into the longevity of repaired versus replaced partially fractured permanent restorations using direct composite restorations. The results suggest that repaired restorations demonstrated comparable longevity to replaced restorations across multiple parameters, including marginal adaptation, anatomic form, surface roughness, marginal staining, and secondary caries development. The meta-estimates favored the repair group for marginal adaptation and secondary caries prevention, suggesting that conservatively done restorations can achieve clinical outcomes similar to complete replacement while preserving more tooth structure.

The significance of marginal adaptation in restorative success cannot be evaded, as it plays a critical role in maintaining the integrity of the restoration and preventing microleakage [20]. The risk ratio analysis indicated that repaired restorations exhibited superior marginal adaptation compared to replaced restorations, with a statistically significant difference. This finding aligns with previous research suggesting that selective removal of defective portions while retaining intact composite material optimizes the adhesive interface, reducing gaps that could otherwise lead to failure [21]. The superior adaptation observed in repaired restorations may be due to the preservation of the already available bonding interface, which reduces stress on the remaining tooth structure and ensures a more stable composite-to-tooth interface.

In terms of anatomic form, the meta-analysis indicated no statistically significant difference between repaired and replaced restorations, suggesting that both approaches perform similarly in maintaining anatomical contour. The ability to maintain the anatomical contour is crucial for functional and aesthetic longevity [22]. Although repaired restorations showed a numerically favorable trend, this was not strong enough to confirm superiority. Clinically, this reinforces that a well-executed repair can be a viable alternative to full replacement, particularly when preservation of existing anatomy is desired, when the restoration is largely intact. Thus, in the absence of significant degradation, repair may be considered a minimally invasive and functionally acceptable strategy.

Surface roughness is another critical factor influencing restoration longevity, as it causes plaque accumulation and, consequently, the risk of secondary caries and periodontal inflammation [23]. While the analysis favored repaired restorations over replacements in terms of surface roughness, the difference was not statistically significant. This finding suggests that both repaired and replaced restorations can achieve similar levels of smoothness when appropriate finishing and polishing techniques are employed. Given that rough surfaces contribute to bacterial adhesion and increased wear over time, this result highlights the importance of meticulous finishing techniques in ensuring restoration longevity regardless of the chosen intervention [24].

Marginal staining, an early indicator of restoration deterioration, was observed to be slightly more prevalent in replaced restorations compared to repaired ones. However, this difference was not statistically significant [25]. Therefore, no definitive conclusion can be drawn regarding the superiority of either approach in preventing marginal staining. While some literature suggests that preserving the existing bonded interface during repair may help reduce microleakage and subsequent discoloration [26], the current evidence from this review does not statistically substantiate this advantage. Further well-powered studies are needed to evaluate whether repair procedures offer aesthetic benefits in terms of marginal staining.

The analysis of secondary caries occurrence revealed a trend suggesting fewer cases in repaired restorations compared to replacements. However, this difference was not statistically significant. As secondary caries remains a primary cause of restoration failure, even a non-significant trend is clinically relevant and warrants further investigation. Theoretically, repair may offer protective advantages by preserving the existing adhesive seal and reducing trauma to surrounding tooth structure, which could help limit bacterial infiltration. Nonetheless, the current evidence does not allow a definitive conclusion, and additional studies with larger sample sizes and longer follow-up are needed to clarify whether repair confers a true advantage in preventing recurrent caries.

Despite the positive outcomes of repaired restorations, variations in study quality and follow-up duration must be noted. While the risk of bias was moderate to high in several studies, the highest levels of bias were observed in domains related to blinding and selection bias. Furthermore, variations in the inclusion and exclusion criteria, as well as differences in restorative protocols, may have influenced the reported longevity outcomes. The findings of this systematic review support the growing body of evidence favoring minimally invasive restorative approaches in cases of partially fractured permanent restorations.

The implications of these findings extend beyond clinical decision-making to create patient-centered care and dental material sustainability. Repairing restorations instead of replacing them not only preserves more tooth structure but also reduces treatment time, cost, and patient discomfort [27]. Additionally, a conservative approach aligns with the principles of minimally invasive dentistry, which prioritizes preserving as much natural tooth structure as possible. Furthermore, repair interventions contribute to sustainable dental practices by minimizing material waste and reducing the environmental impact associated with the frequent replacement of restorations.

## Conclusions

The findings of this systematic review highlight repair as a conservative and effective alternative to full restoration replacement in cases of partially fractured direct composite restorations. The comparable longevity between repaired and replaced restorations, particularly in terms of marginal adaptation and secondary caries prevention, advocates adopting repair strategies whenever feasible. Future studies with standardized methodologies and longer follow-up durations are needed to help refine the evidence base and optimize restorative protocols. Ultimately, embracing repair techniques as a preferred approach can lead to improved patient outcomes, reduced treatment invasiveness, and enhanced long-term success in restorative dentistry.

## Appendices

Sample PubMed search string with complete Boolean operators

Table 4 presents the sample PubMed search strings used in the study.

**Table 4:** PubMed Search String

PubMed Search String	
String 1	((Repair[Title/Abstract]) AND (replacement[Title/Abstract])) AND (composite restoration[Title/Abstract] OR fractured restoration[Title/Abstract])
String 2	"Repair"[Title/Abstract] AND "replacement"[Title/Abstract] AND ("composite restoration"[Title/Abstract] OR "fractured restoration"[Title/Abstract])

## References

[1] Yu H, Zhao Y, Li J (2019). Minimal invasive microscopic tooth preparation in esthetic restoration: a specialist consensus. Int J Oral Sci.

[2] Demarco FF, Collares K, Correa MB, Cenci MS, Moraes RR, Opdam NJ (2017). Should my composite restorations last forever? Why are they failing?. Braz Oral Res.

[3] Bohaty BS, Ye Q, Misra A, Sene F, Spencer P (2013). Posterior composite restoration update: focus on factors influencing form and function. Clin Cosmet Investig Dent.

[4] Mendes LT, Pedrotti D, Casagrande L, Lenzi TL (2022). Risk of failure of repaired versus replaced defective direct restorations in permanent teeth: a systematic review and meta-analysis. Clin Oral Investig.

[5] Al Rabiah A, Zahrah A, Malath T, Al Ebtihal D, Suhaibani Daniyah A, Qahtani Abdullah A (202133). Dental composite restorations repair: a systematic review and meta-analysis. J Pharmaceut Res Int.

[6] Page MJ, Moher D, Bossuyt PM (2021). PRISMA 2020 explanation and elaboration: updated guidance and exemplars for reporting systematic reviews. BMJ.

[7] Luchini C, Stubbs B, Solmi M, Veronese N (2017). Assessing the quality of studies in meta-analyses: advantages and limitations of the Newcastle-Ottawa Scale. World J Meta-Anal.

[8] Corbett MS, Higgins JP, Woolacott NF (2014). Assessing baseline imbalance in randomised trials: implications for the Cochrane risk of bias tool. Res Synth Methods.

[9] Gordan VV, Shen C, Riley J 3rd, Mjör IA (2006). Two-year clinical evaluation of repair versus replacement of composite restorations. J Esthet Restor Dent.

[10] Moncada G, Fernández E, Martín J, Arancibia C, Mjör IA, Gordan VV (2008). Increasing the longevity of restorations by minimal intervention: a two-year clinical trial. Oper Dent.

[11] Gordan VV, Garvan CW, Blaser PK, Mondragon E, Mjör IA (2009). A long-term evaluation of alternative treatments to replacement of resin-based composite restorations: results of a seven-year study. J Am Dent Assoc.

[12] Moncada G, Martin J, Fernández E, Hempel MC, Mjör IA, Gordan VV (2009). Sealing, refurbishment and repair of Class I and Class II defective restorations: a three-year clinical trial. J Am Dent Assoc.

[13] Fernández EM, Martin JA, Angel PA, Mjör IA, Gordan VV, Moncada GA (2011). Survival rate of sealed, refurbished and repaired defective restorations: 4-year follow-up. Braz Dent J.

[14] Fernández E, Martín J, Vildósola P (2015). Can repair increase the longevity of composite resins? Results of a 10-year clinical trial. J Dent.

[15] van de Sande FH, Moraes RR, Elias RV, Montagner AF, Rodolpho PA, Demarco FF, Cenci MS (2019). Is composite repair suitable for anterior restorations? A long-term practice-based clinical study. Clin Oral Investig.

[16] Estay J, Martín J, Viera V (2018). 12 years of repair of amalgam and composite resins: A clinical study. Oper Dent.

[17] Kanzow P, Wiegand A (2020). Retrospective analysis on the repair vs. replacement of composite restorations. Dent Mater.

[18] Santiago SL, Passos VF, Vieira AH, Navarro MF, Lauris JR, Franco EB (2010). Two-year clinical evaluation of resinous restorative systems in non-carious cervical lesions. Braz Dent J.

[19] Cavalheiro CP, Souza PS, Rocha RD, Mendes FM, Braga MM, Raggio DP, Lenzi TL (2020). Choosing the criteria for clinical evaluation of composite restorations: An analysis of impact on reliability and treatment decision. Pesqui Bras Odontopediatria Clín Integr.

[20] Caiafa A, Visser L (2019). Restorative dentistry. Wiggs’s veterinary dentistry: Principles and practice.

[21] Bedran-Russo A, Leme-Kraus AA, Vidal CM, Teixeira EC (2017). An overview of dental adhesive systems and the dynamic tooth-adhesive interface. Dent Clin North Am.

[22] Pizzolotto L, Moraes RR (2022). Resin composites in posterior teeth: clinical performance and direct restorative techniques. Dent J (Basel).

[23] Ereifej NS, Oweis YG, Eliades G (2013). The effect of polishing technique on 3-D surface roughness and gloss of dental restorative resin composites. Oper Dent.

[24] Hilbert LR, Bagge-Ravn D, Kold J, Gram L (2003). Influence of surface roughness of stainless steel on microbial adhesion and corrosion resistance. Int Biodeterior Biodegradation.

[25] Mjör IA, Qvist V (1997). Marginal failures of amalgam and composite restorations. J Dent.

[26] Magalhães CS, Freitas AB, Moreira AN, Ferreira EF (2009). Validity of staining and marginal ditching as criteria for diagnosis of secondary caries around occlusal amalgam restorations: an in vitro study. Braz Dent J.

[27] Sharif MO, Catleugh M, Merry A (2014). Replacement versus repair of defective restorations in adults: resin composite. Cochrane Database Syst Rev.

